# Kynurenine promotes Calcitonin secretion and reduces cortisol in the Japanese flounder *Paralichthys olivaceus*

**DOI:** 10.1038/s41598-023-35222-4

**Published:** 2023-05-29

**Authors:** Takahiro Ikari, Yukihiro Furusawa, Yoshiaki Tabuchi, Yusuke Maruyama, Atsuhiko Hattori, Yoichiro Kitani, Kenji Toyota, Arata Nagami, Jun Hirayama, Kazuki Watanabe, Atsushi Shigematsu, Muhammad Ahya Rafiuddin, Shouzo Ogiso, Keisuke Fukushi, Kohei Kuroda, Kaito Hatano, Toshio Sekiguchi, Ryotaro Kawashima, Ajai K. Srivastav, Takumi Nishiuchi, Akihiro Sakatoku, Masa-aki Yoshida, Hajime Matsubara, Nobuo Suzuki

**Affiliations:** 1 Noto Marine Laboratory, Institute of Nature and Environmental Technology, Kanazawa University, Ogi, Noto-Cho, Ishikawa 927-0553 Japan; 2 Department of Pharmaceutical Engineering, Faculty of Engineering, Toyama Prefectural University, Kurokawa, Toyama 939-0398 Japan; 3 Life Science Research Center, University of Toyama, Sugitani, Toyama 930-0194 Japan; 4 Department of Biology, College of Liberal Arts and Sciences, Tokyo Medical and Dental University, Ichikawa, Chiba 272-0827 Japan; 5 Noto Center for Fisheries Science and Technology, Kanazawa University, Osaka, Noto-Cho, Ishikawa 927-0552 Japan; 6 Department of Clinical Engineering, Faculty of Health Sciences, Komatsu University, Komatsu, Ishikawa 923-0961 Japan; 7 Institute of Nature and Environmental Technology, Kanazawa University, Kakuma, Kanazawa, Ishikawa 920-1192 Japan; 8 Department of Zoology, D.D.U. Gorakhpur University, Gorakhpur, 273-009 India; 9 Bioscience Core Facility, Research Center for Experimental Modeling of Human Disease, Kanazawa University, Takara-Machi, Kanazawa, Ishikawa 920-1192 Japan; 10 School of Science, Academic Assembly, University of Toyama, Gofuku, Toyama 930-8555 Japan; 11 Marine Biological Science Section, Education and Research Center for Biological Resources, Faculty of Life and Environmental Science, Shimane University, Oki, Shimane 685-0024 Japan

**Keywords:** Biochemistry, Physiology

## Abstract

Deep ocean water (DOW) exerts positive effects on the growth of marine organisms, suggesting the presence of unknown component(s) that facilitate their aquaculture. We observed that DOW suppressed plasma cortisol (i.e., a stress marker) concentration in Japanese flounder (*Paralichthys olivaceus*) reared under high-density condition. RNA-sequencing analysis of flounder brains showed that when compared to surface seawater (SSW)-reared fish, DOW-reared fish had lower expression of hypothalamic (i.e., corticotropin-releasing hormone) and pituitary (i.e., proopiomelanocortin, including adrenocorticotropic hormone) hormone-encoding genes. Moreover, DOW-mediated regulation of gene expression was linked to decreased blood cortisol concentration in DOW-reared fish. Our results indicate that DOW activated osteoblasts in fish scales and facilitated the production of Calcitonin, a hypocalcemic hormone that acts as an analgesic. We then provide evidence that the Calcitonin produced is involved in the regulatory network of genes controlling cortisol secretion. In addition, the indole component kynurenine was identified as the component responsible for osteoblast activation in DOW. Furthermore, kynurenine increased plasma Calcitonin concentrations in flounders reared under high-density condition, while it decreased plasma cortisol concentration. Taken together, we propose that kynurenine in DOW exerts a cortisol-reducing effect in flounders by facilitating Calcitonin production by osteoblasts in the scales.

## Introduction

Farmed fish are often reared at high densities, which stresses the fish and compromises their welfare^1,2^. Rearing density influences animal physiology, and increasing density elevates cortisol concentrations in the organisms^1,3^. Cortisol is a stress hormone released into the blood from inter-renal cells of the head kidney^3^. Cortisol synthesis and secretion are regulated by the hypothalamus–pituitary–inter-renal axis, which is involved in the stress response, making cortisol a suitable stress marker^2,3^. In fish, cortisol is an essential hormone that regulates several metabolic activities in the liver and muscles^4–6^. In marine fish, cortisol is also involved in mineral metabolism and osmoregulation^7–9^. However, excess cortisol can induce skeletal muscle atrophy, compromise immune response, and increase energy loss in fish^3,6,10^. Therefore, healthy and stress-free rearing of fish is necessary in fish farming.

Deep ocean water (DOW) is the water found 200 m below the Earth's ocean surface that has three major characteristics, low temperature (approximately 5–9 °C), rich inorganic nutrients (nitrogen, phosphorus, and silicate), and clean water (minimal to no bacterial activity and less phytoplankton photosynthesis)^11,12^. The growth of seaweeds^13,14^ and shrimp^15^ is reported to be improved by rearing them in DOW compared with the growth of rearing them in surface seawater (SSW). For example, the weight gain rate of the brown alga *Sargassum fusiforme* in DOW is higher than that in SSW^14^. Similar to *S. fusiforme*, juvenile sporophytes of *Eisenia arborea* and *Eisenia cava* grow faster in DOW than in SSW^13^. The deep-sea pelagic shrimp *Sergia lucens* grow for 185 and 17 days in DOW and SSW, respectively^15^. Therefore, DOW has some physiologically significant effects on aquatic organisms.

The health effects of mineral ions found in DOW on humans have been studied^11,16,17^. In addition to minerals, the DOW has been reported to contain organic compounds, which have gained attention. For example, a diterpene, sandaracopimarinol, produced by the Japanese cedar (*Cryptomeria japonica*) has been detected in the DOW (687 m) of Suruga Bay, Japan^18^. This finding suggests that terrestrial organic compounds are carried through rivers and deposited in DOW. These compounds may be present in DOW, either from terrestrial sources or from discharges of fish and other aquatic organisms. This study focused on indole compounds, which are ubiquitous in various animals and plants and have a bioactivity of stress-reducing effect^19–25^. For example, tryptophan supplementation in rainbow trout (*Oncorhynchus mykiss*), a fish that exhibits dominant behavior, suppresses aggressive behavior and reduces plasma cortisol concentration^23^. In addition, the indole compounds melatonin and serotonin, which are metabolites of tryptophan, are involved in the stress response^21,22^.

Kynurenine, a metabolite of tryptophan, has immune and inflammatory responses and is involved in stress responses in the brain^24,25^. Kynurenine is generally produced in response to stress and inflammation. The conversion of tryptophan to kynurenine requires an enzyme known as indoleamine 2,3-dioxygenase (IDO)^26^. IDO is expressed mainly in immune and neuronal cells and is induced by cortisol^27^, thereby linking the cortisol and kynurenine pathways. However, whether kynurenine is involved in the regulation of cortisol secretion remains unknown.

We compared the plasma cortisol, mineral, and component concentrations as well as gene expression profiles of brains between the Japanese flounders *Paralichthys olivaceus* reared under high-density condition in either SSW or DOW. The comparison indicated that DOW suppresses plasma cortisol concentration in the flounder reared under high-density condition and further that kynurenine, an indole compound presented in DOW but not in SSW, is involved in the cortisol-reducing effects of DOW. In addition, we provided evidence that kynurenine activates osteoblasts in the scales to produce Calcitonin, which is then transported to the brain and suppresses the cortisol secretion.

## Results

### Cortisol concentrations in flounders reared under density stress in SSW or DOW for 5 and 10 days

**Figure 1:**
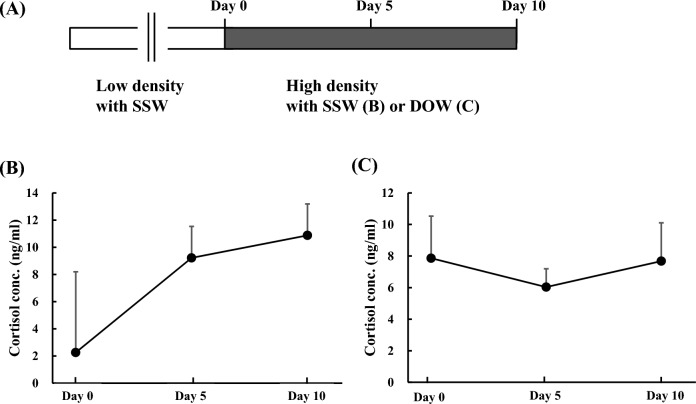
Changes in the plasma cortisol concentrations in flounder at 5 and 10 days after rearing with surface seawater (SSW) or deep ocean water (DOW). (**A**) Schematic of experimental setup for Fig. 1B and C. Fish were kept under low density condition with SSW, then transferred to the high density condition with SSW (**B**) or DOW (**C**). Blood analytical samples were prepared from fish at the indicated points after the transfer to the high density condition. Black and white bars represent low density and high density conditions, respectively. Flounder in SSW and DOW, n = 7.

Plasma cortisol concentration was measured in flounders that had been transferred from a large to a small aquarium and reared under 6.25 times density stress in either SSW or DOW (Fig. 1A). Plasma cortisol concentrations in flounders reared in SSW increased significantly (*P* < 0.01; Fig. 1B), while that of flounders reared in DOW did not (*P* = 0.88; Fig. 1C).

### Changes in plasma components of flounders reared under density stress with SSW or DOW

**Figure 2:**
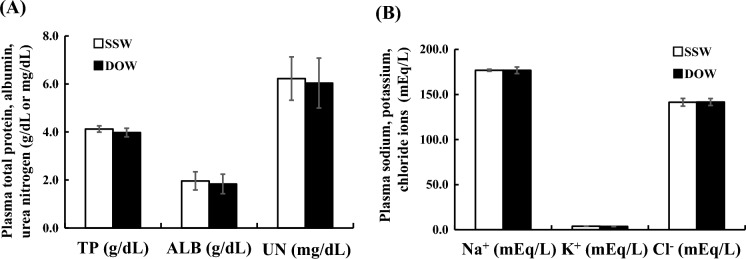
Changes in the plasma component (total protein: *TP*, albumin: *ALB*, and urea nitrogen: *UN*) and mineral (sodium ion: Na^+^, potassium ion: K^+^, chloride ion: Cl^−^) concentrations at 10 days after rearing flounder with SSW or DOW. SSW: n = 7; DOW: n = 7.

**Figure 3:**
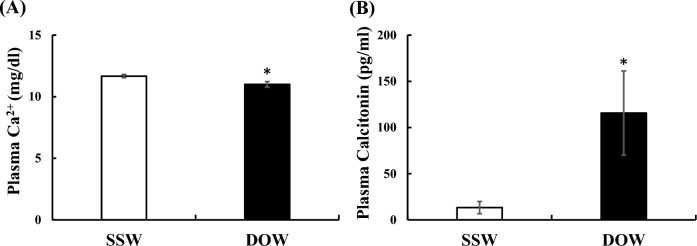
Changes in the plasma calcium ion (Ca^2+^) (mg/dl) (**A**) and Calcitonin (pg/ml) (**B**) concentrations at 10 days after rearing flounder with SSW or DOW. SSW: n = 7; DOW: n = 7, *P < 0.05.

The total protein (TP), albumin (ALB), and urea nitrogen (UN) concentrations in the plasma of flounders at 10 days after rearing in SSW or DOW showed no significant differences (Fig. 2A). Similarly, the concentrations of plasma Na^+^, K^+^, and Cl^−^ ions in flounders reared in SSW and DOW did not significantly differ from one another (Fig. 2B). In contrast, the plasma Ca^2+^ concentrations in flounders reared in DOW was significantly lower than that of flounders reared in SSW (Fig. 3A), although Ca^2+^ concentrations in SSW and DOW did not differ (Table S1). Furthermore, plasma Calcitonin concentrations of flounders reared in DOW were significantly higher than those of flounders reared in SSW (Fig. 3B).

### RNA-sequencing analysis of the brain and skin of flounders reared under density stress in SSW or DOW

**Figure 4:**
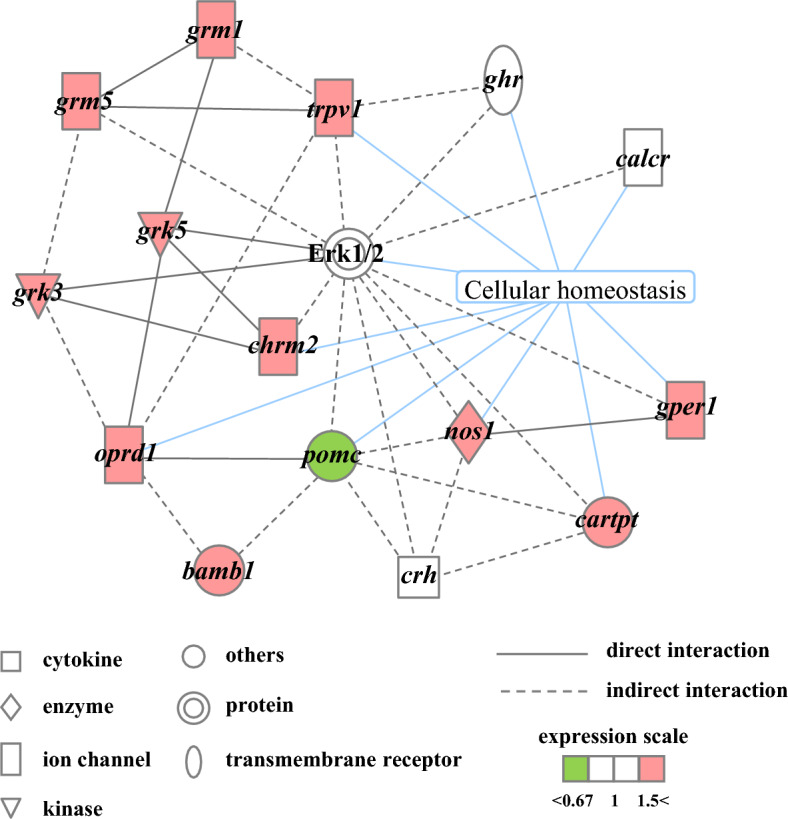
Gene network analysis of flounder brain: Analysis of Calcitonin's analgesic action and stress response. Differentially expressed genes in the brain of flounder reared with DOW were compared to those in the brain of flounder reared with SSW using the Ingenuity Pathway Analysis tools. The network is represented graphically with nodes (genes) and edges (the biological associations between the nodes). *bambi*: *bmp and activin membrane bound inhibitor*, *calcr*: *calcitonin receptor*, *cartpt*: *cart prepropeptide*, *chrm2*: *cholinergic receptor muscarinic 2*, *crh*: *corticotropin releasing hormone*, Erk1/2: Extracellular signal-regulated kinase, *ghr*: *growth hormone receptor*, *gper1*: *G protein-coupled estrogen receptor 1*, *grk3*: *G protein-coupled receptor kinase 3*, *grk5*: *G protein-coupled receptor kinase 5*, *grm1*: *glutamate metabotropic receptor 1*, *grm5*: *glutamate metabotropic receptor 5*, *nos1*: *nitric oxide synthase 1*, *oprd1*: *opioid receptor delta 1*, *pomc*: *proopiomelanocortin*, *trpv1*: *transient receptor potential cation channel subfamily V member 1*.

RNA-sequencing analysis showed lower expression levels of *corticotropin-releasing hormone* (*crh*)^28^ and *pro-opiomelanocortin* (*pomc*)^29^ in the brains of flounders reared in DOW compared with those in the brains of flounders reared in SSW (Table S2). Genes related to the analgesic action (Table S3) were extracted together with *crh*, *pomc*, and *calcitonin receptor* in the brain of flounders and further analyzed using Ingenuity Pathway Analysis tools. The results of pathway analysis are shown in Fig. 4, and the data are shown in Table S3. The genes involved in analgesia formed a network with *pomc*, *crh*, and the *calcitonin receptor* (*calcr* in Fig. 4).

### Direct effects of SSW and DOW on osteoblasts of goldfish scales

**Figure 5:**
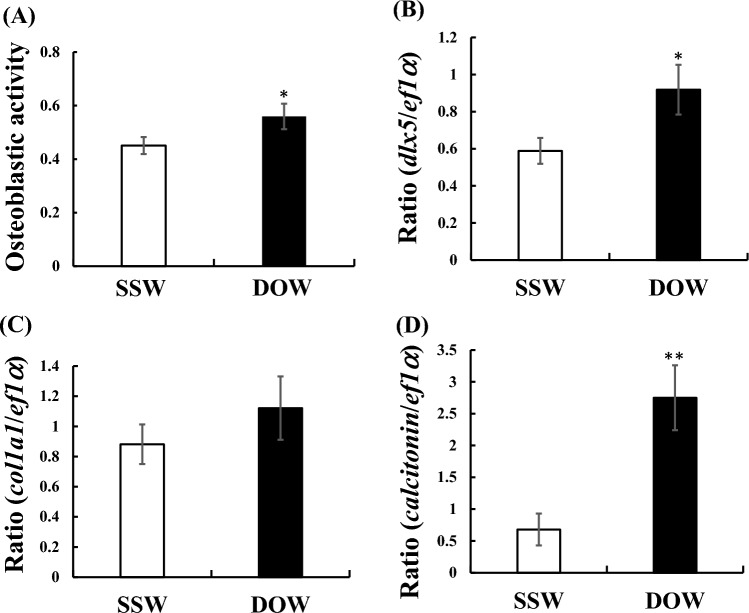
Effects of SSW and DOW on osteoblastic activity (**A**), mRNA expression of osteoblastic markers (**B**, *dlx5*), (**C**, *collagen type I alpha 1*: *col1a1*), and (**D**, *calcitonin*) in fish scales. SSW: n = 8; DOW: n = 8, *P < 0.05; **P < 0.01.

RNA-sequencing analysis of genes expressed in the epidermis, including the scales of flounder, revealed elevated expression levels of *calcitonin* mRNA (Table S4). Therefore, we investigated the effects of DOW and SSW on the osteoblasts of flounder scales by adding DOW or SSW to the culture medium at a rate of 20% followed by incubation for 24 h. We observed higher osteoblast activity in goldfish (*Carassius auratus*) scales cultured with DOW compared with those cultured with SSW in the medium (Fig. 5A). mRNA expression levels of the osteoblastic marker (*dlx5*, Fig. 5B; *col1a1*, Fig. 5C) were also higher in fish scales cultured with DOW. For example, the expression level of *dlx5* was significantly (*P* < 0.05) higher in fish scales cultured with DOW compared with that in fish scales cultured with SSW (Fig. 5B). Furthermore, *calcitonin* mRNA expression levels in fish scales treated with DOW were significantly higher than those in fish scales treated with SSW in the medium (Fig. 5D). The data of the increased expression of *calcitonin, dlx5*, and *col1a1* mRNA of flounders reared in DOW compared with those of flounders in SSW (Table S4) were consistent with the data obtained from goldfish scales.

### Indole compounds in SSW and DOW

We reported that an indole compound melatonin promoted Calcitonin secretion by scale osteoblasts^30^. Since there is no difference in trace minerals between DOW and SSW (Table S5), we speculated that an indole compound facilitating Calcitonin secretion exists in DOW but not in SSW. Accordingly, we analyzed for their indole compound concentrations of DOW and SSW (Table 1). The compounds N-acetyl-N-formyl-5-methoxykynuramine, kynuramine, and indole-3-acetic acid were detected in SSW, whereas kynurenine and indole-3-acetic acid were detected in DOW. Among these compounds, kynurenine was found only in DOW.

**Table 1:** Indole compounds (pg/L) contained in SSW and DOW.

	SSW	DOW
MEL	–	–
AMK	–	–
AFMK	7.85	–
5HT	–	–
NAS	–	–
HaMT	–	–
Kynuramine	292.01	–
Kynurenine	–	62.14
5MTP	–	–
IAA	9.6	3.5

*Mel* melatonin; *AMK* N-acetyl-5-methoxykynuramine; *AFMK* N-acetyl-N-formyl-5-methoxykynuramine; *5HT* 5-hydroxytryptamine; *NAS* N-acetyl serotonin; *HaMT* 6-hydroxymelatonin; *5MTP* 5-methoxytryptophan; *IAA* Indole-3-acetic acid.

### Stress-reducing effect of kynurenine in flounders

**Figure 6:**
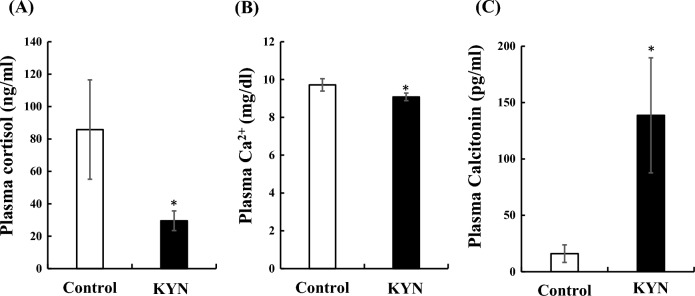
Effects of kynurenine on the concentrations of plasma cortisol (**A**), Ca^2+^ (**B**), and Calcitonin (**C**) at 5 days after rearing founder with or without kynurenine (10^−6^ M). Each n = 10, *P < 0.05.

The effect of kynurenine on plasma cortisol concentrations of flounders reared in artificial seawater under density stress was investigated. After 5 days, plasma cortisol concentrations in kynurenine-treated flounders were significantly lower than those in control (Fig. 6A). Furthermore, the plasma Ca^2+^ concentrations in the kynurenine-treated flounders were significantly lower than that in the control flounders (Fig. 6B), whereas the plasma Calcitonin concentrations in the kynurenine-treated flounders were significantly higher than those in control (Fig. 6C).

### Effect of kynurenine on *calcitonin* mRNA expression in osteoblasts of fish scales

**Figure 7:**
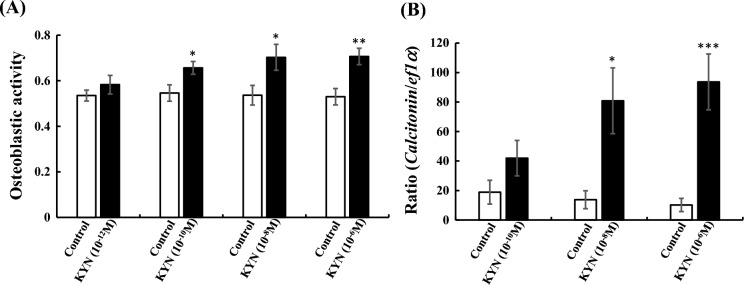
Effects of kynurenine on osteoblastic activity (**A**) and *calcitonin* mRNA expression (**B**) in goldfish scales. Osteoblastic activity and *calcitonin* mRNA expression were assessed using an independent sample *t* test or paired *t* test, respectively. Each n = 8, *P < 0.05; **P < 0.01; ***P < 0.001.

Osteoblastic activity and *calcitonin* mRNA expression in osteoblasts were studied using the scales by in vitro assay to examine *calcitonin* mRNA expression in osteoblasts by kynurenine treatments. Figure 7A shows that the activity of osteoblasts increased significantly after incubating fish scales in a medium supplemented with kynurenine (10^−10^, 10^−8^, and 10^−6^ M). Similarly, *calcitonin* mRNA expression levels increased significantly with the addition of kynurenine (10^−8^ and 10^−6^ M) (Fig. 7B).

## Discussion

**Figure 8:**
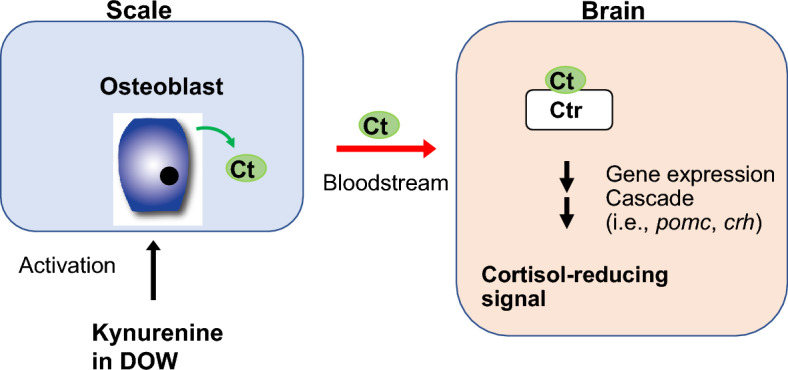
A proposed model of the mechanism underlying kynurenine-mediated suppression of cortisol secretion. *Ct*: Calcitonin, *Ctr*: Calcitonin receptor, *DOW*: Deep ocean water, *crh*: *corticotropin-releasing hormone*, *pomc*: *pro-opiomelanocortin*.

DOW has been known to positively influence the growth of seaweed^13,14^ and shrimp^15^. We have identified another positive influence of DOW, namely its cortisol-reducing effect on flounders. Our results provide several lines of evidence that DOW activates osteoblasts in the scales and promotes the production of Calcitonin, which has an important cortisol-reducing effect (Fig. 8). It is known that fish scales possess vascular tissue, including blood vessels^31,32^. In addition, Calcitonin has been shown to cross the blood–brain barrier in rats^33^. Given these findings, we propose that the Calcitonin produced by osteoblasts in the scales is transported to the brain via the bloodstream (Fig. 8). We further speculate that Calcitonin regulates the expression of genes involved in the regulation of cortisol secretion in the brain—such as *crh* and *pomc* —thereby resulting in lower blood cortisol concentration (Fig. 8).

Our analysis of gene expression profiles in the brains of flounders identified a candidate gene network involved in the *calcitonin*-dependent suppression of cortisol secretion. In this network, Calcitonin was thought to bind to the Calcitonin receptor and to thereby transduce a signal inhibiting the expression of the *crh* and *pomc* genes (Fig. 4). This signal transduction was likely mediated by several molecules, including *opioid receptor delta 1* (*oprd1*), which directly associates with *pomc*. Opioid receptors are usually G-protein coupled receptors that bind to neurotransmitters and opioids outside the cell and trigger a response via G proteins inside the cell^34^. Moreover, opioids bound to opioid receptor delta molecules, thereby suppressing the pain response, exerting an analgesic effect^35^. Thus, the involvement of Oprd1 with the Calcitonin-mediated suppression of cortisol in the brain is consistent with the fact that Calcitonin also exerts an analgesic function in the central modulation of pain perception^36,37^.

In the current study, kynurenine activated osteoblasts and promoted their production of *calcitonin* in cultured fish scales (Fig. 7). In addition, it increased the blood Calcitonin concentrations of flounders in vivo in a manner similar to the phenomenon induced by DOW (Fig. 6). Based on these findings, we propose that kynurenine in DOW is a factor responsible for the DOW-induced activation of osteoblasts in the scales and the resulting Calcitonin production (Fig. 8); this therefore explains the cortisol-reducing effect of DOW on flounders. Kynurenine is metabolized to quinolinic acid (QA) via the other metabolites^26,38^. QA induces the release and inhibits the reuptake of glutamate causing excitotoxicity^39^, and this is hypothesized to act as a link between chronic stress, depression, and inflammation^40^. On the other hand, kynurenine is also metabolized to kynurenic acid, which is considered to be neuroprotective^41^. The current study revealed a novel mechanism in which kynurenine induces Calcitonin production in osteoblasts and increases the expression of analgesia genes in the brain to reduce blood cortisol levels (Fig. 8).

Previous study evaluated effect of stocking density on expression level of a stress marker gene *Hsp70* in muscle of rainbow trout (*Oncorhynchus mykiss*)^42^ and sea bass (*Dicentrarchus labrax, L.*)^43^. In rainbow trout, the *Hsp70* expression was found to increase under the high density conditions (24 and 44 kg/m^3^) compared to that under the low density condition (12 kg/m^3^). In addition, in sea bass, the *Hsp70* expression was found to significantly over-express under the biomass of 100 kg/m^3^. Myosin is a ubiquitous motor protein and an ideal indicator for growth studies and *myosin heavy chain* (*mhc*) mRNA levels are known as a possible marker of trout muscle growth^44^. On other hand, myostatin (Mstn), is considered as a negative regulator of fish muscle growth^45,46^. In rainbow trout, expression of *mhc* decreased significantly under the high density condition (44 kg/m^3^) compared to that under the low density condition (12 kg/m^3^), while *mstn-1a* increased. Therefore, the density (24 kg/m^3^) we used is reasonable. In addition, in rainbow trout, a species with typically dominant behavior, tryptophan supplementation suppresses aggressive behavior and induces lower plasma cortisol concentration compared to unsupplemented rainbow trout^23^. This report is consistent with our findings, and can be attributed to the metabolism of tryptophan to kynurenine^24,25^, which thereafter exerts a stress-reducing effect.

Next, we also found that the plasma Ca^2+^ concentration was significantly lower in fish reared in DOW than in the fish reared in surface seawater (SSW), although these groups did not differ in their concentrations of plasma Na^+^, K^+^, or Cl^−^ after ten days (Figs. 2B and 3A). Similarly, we also did not observe significant differences in plasma TP, ALB, and UN concentration between groups (Fig. 2A). The lower plasma Ca^2+^ levels in fish reared in DOW might be due to Calcitonin eliciting hypocalcemic activity^37^. In addition, flounders reared in DOW had significantly higher plasma Calcitonin concentration compared to flounders reared in SSW (Fig. 3B). Calcitonin is a hypocalcemic hormone that regulates plasma Ca^2+^ concentration to a constant level of approximately 10 mg/dL in teleosts and mammals^37^. Calcitonin is known to suppress osteoclastic activity in fish^47^ and mammals^37^ to reduce plasma Ca^2+^ concentration.

Aquaculture stresses fish because they are typically reared at high densities. Under these conditions, measures to reduce stress should be considered to improve fish welfare^1,2^. Furthermore, because excess cortisol induces skeletal muscle atrophy, compromises immune response, and increases energy waste in fish^3,6,10^, methods without stressing them are necessary. Finally, based on our results, the use of kynurenine or DOW for rearing fish may solve problems related to stress, and thereby promote fish welfare.

## Conclusion

In this study we demonstrated that DOW reduces plasma cortisol (i.e., a stress marker) concentration in flounders reared under high-density condition. The cortisol-reducing action of DOW is attributed to Calcitonin produced by osteoblasts in the scales. In particular, we propose that Calcitonin produced in the scales is transported to the brain, where it contributes to the expression of genes involved in cortisol secretion. In addition, the indole component kynurenine in DOW was found to be responsible for osteoblast activation and the resulting production of Calcitonin, contributing to suppression of cortisol secretion. Overall, our results suggest a novel biological function of kynurenine, which can be used to reduce stress in fish reared in aquaculture environments.

## Methods

### Statements on the ethical treatment of animals

This study was carried out in strict accordance with the recommendations in the ethical guidelines of Kanazawa University. All experimental protocols in this study were approved by the Animal Welfare Committee of Kanazawa University. In addition, all experimental protocols in this study were in strict accordance with the ARRIVE guidelines 2.0^48^. We made maximum efforts to avoid causing pain and distress to experimental animals. When biological samples were collected from the animals, pain was minimized by anesthesia preoperatively. Particular attention was paid to the use of anesthesia to alleviate discomfort.

### Animals

Flounders (*P. olivaceus*) were purchased from Marinetech Co. Ltd. (Aichi, Japan) and they were cultivated. Goldfish (*C. auratus*) purchased from Higashikawa Fish Farm (Yamatokoriyama, Japan) were used to examine the effects of the bioactive substance in DOW on fish scales. The DOW was pumped from 320 m at a facility (Aquas Noto, Ishikawa prefecture, Japan). After the aeration of DOW in an aquarium, the dissolved oxygen was maintained at approximately 7 mg/L. SSW was pumped from Tsukumo Bay (Noto Peninsula, Ishikawa Prefecture), where our marine laboratory is located, and was stored in an aquarium with bubbling air. The dissolved oxygen of SSW was also maintained at approximately 7 mg/L. A system of DOW and SSW rearing was used in which wastes were filtered out in a filtration tank. Each of fish was reared at a density of 24 kg/m^3^ in a single aquarium (60 cm × 25 cm × 30 cm). Fish were collected sequentially from the same tank.

Flounders acclimated in SSW at 20 °C for 1 week (mean density: 241 g/62.5 L; 200 cm × 100 cm × 65 cm) were used. Meanwhile, goldfish acclimated in freshwater at 25 °C for approximately 1 week were used for in vitro experiments. To prepare the biological samples for analyses, the fish were anesthetized with a 0.04% 2-phenoxyethanol (FUJIFILM Wako Pure Chemical Corporation, Osaka, Japan) or MS-222 (Sigma-Aldrich, Inc. St. Louis, MO, USA) and then were decapitated. MS-222 was neutralized with sodium bicarbonate.

### Rearing of marine teleost in DOW or SSW

The density during acclimation was 3.85 kg/m^3^. After acclimation, flounders were reared in aquaria (mean density: 241 g/62.5 L; 200 cm × 100 cm × 65 cm) for 1 week. The flounders were grouped in separate aquaria containing either SSW or DOW (n = 7) at mean densities of 24 g/L, which was 6.25 times the stress density. The value for density stress threshold was defined based on the previous study^42^. Fish were reared with SSW or DOW for 10 days at 20 ± 1 °C under a 12 h:12 h light–dark cycle. Each day, 1/10th of the volume of water in each aquarium was replaced. The fish were fed with artificial feed (4% body weight; Otohime, Marubeni Nisshin Feed Co., Ltd., Tokyo, Japan) once every morning. Prior to rearing in the small aquaria, the fish were anesthetized with a 0.04% 2-phenoxyethanol (FUJIFILM Wako Pure Chemical Corporation) to avoid causing pain, and their blood was sampled from caudal vessels using a heparinized syringe. The same blood sampling procedure was followed at 5 and 10 days after rearing. The collected blood was transferred into a 1.5-mL tube and then centrifuged at 5200 × g for 5 min. The separated plasma was immediately frozen and kept at − 80 °C until use.

### Measurement of plasma cortisol and Calcitonin

A five-fold volume of diethyl ether was added to the plasma sample, and the ether layer was evaporated with nitrogen gas. The dried sample was dissolved in assay buffer (50 mM H_3_BO_3_, 0.2% bovine serum albumin, 0.01% thimerosal, pH 7.8), and the plasma cortisol concentration was measured using an enzyme-linked immunosorbent assay (ELISA) kit (Cosmo Bio Co. Ltd., Tokyo, Japan).

The competitive ELISA procedure for Calcitonin was performed as described by Suzuki^49^. In separate glass tubes, 250 μL of diluted antibody (400,000 times) with a diluting solution (10 mM phosphate-buffered solution containing 0.1% bovine serum albumin and 0.1% NaN_3_, pH 7.4) was pre-incubated with the same volume of serially diluted synthetic salmon Calcitonin (6.25, 12.5, 25, 50, 100, 200, 400, and 800 pg/mL) or plasma samples diluted three times with a diluting solution for 3 days at 4 °C. Thereafter, salmon Calcitonin-coated plates were washed four times with a washing buffer solution (10 mM phosphate-buffered solution containing 0.05% Tween 20). Aliquots (100 μL) of the pre-incubated synthetic salmon Calcitonin or plasma samples were transferred to the coated plates and incubated at 4 °C for 24 h. After washing four times with the washing buffer solution, color development was performed. Eight milligrams of *o*-phenylenediamine dihydrochloride was added to 12 mL 0.1 M citric acid and 0.2 M Na_2_HPO_4_ solution (pH 4.5) containing 2.4 μL of hydrogen peroxide (30% solution, FUJIFILM Wako Pure Chemical Corporation). Aliquots of 100 μL of the solution were added and incubated in wells with constant agitation at room temperature for 5 min. After incubation, the reaction was stopped, followed by the addition of 50 μL 3 N sulfuric acid. The optical density of the reacted triplicate samples in plates was measured at 492 nm using a microplate reader (MTP-500, Corona Electric Co. Ltd., Tokyo, Japan). After color development, a regression curve for synthetic salmon Calcitonin was drawn using Excel (Microsoft Office, Microsoft Corporation, San Francisco, CA, USA) to determine the concentration of the sample.

The specificity of the antibody was checked using peptide hormones 1–34 bovine PARATHYROID HORMONE and human CALCITONIN GENE-RELATED PEPTIDE^49^.

### Measurement of plasma components and seawater (DOW and SSW)

Ten days after rearing flounders in SSW or DOW, their plasma samples were analyzed by Oriental Yeast Co., Ltd. (Tokyo, Japan), where plasma TP, ALB and UN were measured using L-type Wako TP, L-type Wako ALB-BCP, and L-type Wako UN-V kits (FUJIFILM Wako Pure Chemical Corporation), respectively. In addition, plasma levels of Na^+^, Cl^−^, and K^+^ were measured by an ion electrode method with a Hitachi 7180 automatic analyzer (Hitachi High Technologies Corporation, Tokyo, Japan). The plasma Ca^2+^ concentration was determined using the Ca II assay kit (Shino-Test Corporation, Tokyo, Japan). Mineral components, such as Na^+^, Cl^−^, K^+^, and Ca^2+^, in DOW and SSW were measured as described above.

Trace minerals in flounder plasma (pooled sample for two individuals) were also analyzed. After the removal of protein by 10% trifluoroacetic acid and centrifugation, the supernatant was passed through a 0.22-μm filter. The filtrate was used to measure the trace minerals in their plasma by inductively coupled plasma mass spectrometry (X7, Thermo Inc., Waltham, MA, USA)^50^.

### Analysis of mRNA expression in the brain and skin after rearing flounders in SSW or DOW

Flounders anesthetized with 0.04% 2-phenoxyethanol and reared in either SSW (n = 10) or DOW (n = 10) were dissected after decapitation, and the brain and skin samples were collected. The separated samples were placed in RNA*later* (Sigma-Aldrich Inc., St. Louis, MO, USA) and frozen at − 80 °C for mRNA analysis.

Total RNA was extracted using a NucleoSpin RNA II kit (Takara Bio Inc., Otsu, Japan) following the manufacturer’s instructions. Genomic DNA was removed from the extract using an RNase-Free DNase Set (Takara Bio Inc.). Total RNA extracted from 10 individuals was diluted to 2 nM, mixed in equal amounts (10 µL each), and used as one sample for RNA-sequencing. RNA-seq libraries for directional paired-end reads (100-bp paired-end) were constructed from flounder mRNAs using TruSeq RNA Sample Prep Kit v2 and sequenced on a HiSeq 2500 platform (Illumina, San Diego, CA, USA) using cluster generation. Quality control on raw reads was performed using FastQC ( http://www.bioinformatics.babraham.ac.uk/projects/fastqc/ )^51^, and high-quality reads (> Q20 and > 36 bp) without adaptors were extracted using Trimmomatic^52^. Further, a transcriptome was assembled de novo with the filtered reads using Trinity with default parameters to establish the reference sequence of flounder transcripts^53^. Sequence reads were pseudomapped to the reference sequence using kallisto with default parameters; the pseudoalignment rates were approximately 92%^54^. The assembled sequences were annotated using Blastx and Blastn against the NCBI database. The mapped and annotated read counts were normalized using the Transcripts Per Million reads method by kallisto. Gene ontology, including biological processes, molecular functions, and gene networks of sequence data (accession no. DRA015483) were analyzed using Ingenuity Pathway Analysis tools (Qiagen, Venlo, Netherlands)^55^.

### Direct effects of SSW and DOW on the osteoblasts of fish scales

The direct effects of SSW and DOW on fish scales were analyzed using an in vitro assay system consisting of goldfish scales^47,56^. Teleost scales regenerate after being removed. The osteoblastic activity in regenerating scales is considerably higher than that in normal scales^57–59^. Therefore, we used regenerating scales to examine the influence of SSW and DOW on osteoblasts.

Goldfish (n = 24) were anesthetized with MS-222 (Sigma-Aldrich, Inc.) solution neutralized with sodium bicarbonate, and a total of 32 scales were collected from both sides of each goldfish. The scales comprised eight from each side of one row of scales above and below the lateral line. Ten days after the removal of the scales, the goldfish were anesthetized again, and the regenerating scales were removed and used to investigate the influence of SSW and DOW on osteoblastic enzymatic activity and mRNA expression. These regenerating scales were incubated at 15 °C for 24 h in Leibovitz’s L-15 Medium (Thermo Fisher Scientific Inc., Grand Island, NY, USA) containing 20% of either SSW or DOW and 1% antibiotic mixture (10,000 units/mL of penicillin and 10,000 µg/mL of streptomycin; Thermo Fisher Scientific, Inc.). After incubation, some scales were analyzed for alkaline phosphatase, which is an osteoblastic marker^47,56,57^, while the other scales were placed in RNA*later* (Sigma-Aldrich Inc.) and frozen at − 80 °C for mRNA analysis.

Total RNAs were extracted from goldfish scales using NucleoSpin RNA II kit (Takara Bio Inc.). Genomic DNA was removed from the extract using RNase-Free DNase Set (Takara Bio Inc.). Complementary DNA was synthesized using a PrimeScript II 1st strand cDNA Synthesis Kit (Takara Bio Inc.). The gene-specific primers for amplifying *dlx5*, *col1a1*, and *calcitonin* are listed in Table S6. The *elongation factor1α* (*ef1α*) cDNA was amplified as a housekeeping gene using the primer set listed in Table S6. PCR amplifications were performed using a real-time PCR apparatus (Mx3000p; Agilent Technologies, Santa Clara, CA, USA). The mRNA levels of *dlx5*, *col1a1*, and *calcitonin* were normalized to the *ef1α* mRNA level^60^.

### Analysis of indole compounds in SSW and DOW

DOW and SSW (each 5 L) were adsorbed on an ultrafiltration system (3 M 2215 C18 Empore Extraction Disks, GL Sciences Inc., Tokyo, Japan) with a quantity of ultrafiltrated water and eluted with 10 mL of ethanol and 30 mL of benzene. The eluted solution was dried up by nitrogen gas and analyzed by liquid chromatography–tandem mass spectrometry (LC–MS/MS, LCMS-8050; Shimadzu Co., Kyoto, Japan). Each of the dried samples was resuspended in 100 μL of Milli-Q water and centrifuged. The supernatant was passed through a 0.22-µm filter, and the filtrate was analyzed by LC–MS/MS. The indole compounds in SSW or DOW were simultaneously analyzed as described ^61^.

### Stress-reducing effect of kynurenine in flounder

Flounders were reared in artificial sea water (SEALIFE, Marinetech Co. Ltd.) with or without kynurenine (10^−6^ M; FUJIFILM Wako Pure Chemical Corporation) at a stress density described above. Flounders were kept directly in the aquarium without a filtration tank. The water was changed daily. Five days after rearing, blood samples were collected from the caudal vessels of anesthetized (0.04% 2-phenoxyethanol) fish using a heparinized syringe. The collected blood was transferred into a 1.5 mL tube, and the tube was centrifuged at 5200 × g for 5 min. The separated plasma was immediately frozen and stored at − 80 °C until use. The plasma Cortisol, Ca^2+^, and Calcitonin concentrations were measured as described above.

### Effect of kynurenine on the osteoblasts of fish scales

The effects of kynurenine on the osteoblasts of fish scales were investigated using an in vitro assay system with regenerating goldfish scales. The regenerating scales were prepared as described above, and placed in Leibovitz’s L-15 Medium (Thermo Fisher Scientific Inc.) containing 10^−12^, 10^−10^, 10^−8^, and 10^−6^ M kynurenine. The osteoblasts’ activities of the scales in the same individual were similar in goldfish^58^. Scales for the experiment with each dose were obtained from the same fish. The half of collected scales were then used for the control experiment and the other half for the kynurenine-treated experiment. The scales were incubated at 15 °C for 24 h, and the osteoblastic activity was measured as described above.

The *calcitonin* mRNA expression levels of the control and kynurenine-treated groups were compared within one individual goldfish and investigated by incubating regenerating scales in a medium supplemented with 10^−10^, 10^−8^, and 10^−6^ M kynurenine at 15 °C for 24 h. After incubation, the scales were placed in RNA*later* (Sigma-Aldrich Inc.) and frozen at − 80 °C for mRNA analysis.

Total RNA was extracted using NucleoSpin RNA II kit (Takara Bio Inc.) following the manufacturer’s instructions. Genomic DNA was removed from the extract using an RNase-Free DNase Set (Takara Bio Inc.). Complementary DNA was synthesized using a PrimeScript II 1st strand cDNA Synthesis Kit (Takara Bio Inc.). The expression of *calcitonin* mRNA was examined using real-time PCR methods as described above.

### Statistical analysis

All results were expressed as the mean ± standard error. Based on the Shapiro–Wilk test, cortisol levels at each time point did not follow a normal distribution. Therefore, cortisol concentration was compared by a nonparametric Friedman test in R^62^. The statistical significance between the control and experimental groups was assessed using an independent sample t-test or paired t-test. In all cases, the selected significance level was *P* < 0.05.

## Supplementary Information

Supplementary Information 1.

Supplementary Information 2.

## Data Availability

The raw sequence reads were deposited at the DNA Data Bank of Japan (DDBJ) under the DDBJ Sequence Read Archive (DRA) accession no. DRA015483 (https://ddbj.nig.ac.jp/resource/sra-submission/DRA015483).
