# Supplementary material for: Kynurenine promotes Calcitonin secretion and reduces cortisol in the Japanese flounder Paralichthys olivaceus
Source: Sci Rep. 2023 May 29;13:8700. doi: 10.1038/s41598-023-35222-4 (PMC10227051; doi:10.1038/s41598-023-35222-4)
Supplement: Supplementary file 1 — Supplementary Information 1. [file 41598_2023_35222_MOESM1_ESM.docx]

**Supplementary data for**

**Kynurenine reduces cortisol and promotes calcitonin secretion in Japanese flounder *Paralichthys olivaceus***

**Takahiro Ikari, Yukihiro Furusawa, Yoshiaki Tabuchi, Yusuke Maruyama, Atsuhiko Hattori, Yoichiro Kitani, Kenji Toyota, Arata Nagami, Jun Hirayama, Kazuki Watanabe, Atsushi Shigematsu, Muhammad Ahya Rafiuddin, Shouzo Ogiso, Keisuke Fukushi, Kohei Kuroda, Kaito Hatano, Toshio Sekiguchi, Ryotaro Kawashima, Ajai K. Srivastav, Takumi Nishiuchi, Akihiro Sakatoku,** **Masa-aki Yoshida, Hajime Matsubara, and Nobuo Suzuki**

**This file includes:**

Supplementary Table S1, S2, S4, S5, and S6

*Supplementary Table S3 is included in the separate Excel file due to the fact that it contains a large amount of data.

Table S1 Mineral concentrations (sodium ion: Na^+^; chloride ion: Cl^-^; potassium ion: K^+^; calcium ion: Ca^2+^) of SSW and DOW.

|  | **Na^+^ (mEq/L)** | **K^+^(mEq/L)** | **Cl^-^**  **(mEq/L)** | **Ca^2+^ (mg/dL)** |
| --- | --- | --- | --- | --- |
| **SSW** | **470** | **9.9** | **470** | **41** |
| **DOW** | **470** | **10** | **470** | **41** |
|  |  |  |  |  |

Table S2 Expression values (in pooled samples) for the genes involved in cortisol secretion in the brains of flounder reared in SSW and DOW.

| **TPM** | **SSW** | | **DOW** |
| --- | --- | --- | --- |
| ***crh*** | **7.53722** | | **5.6345** |
| ***pomc*** | **1.053** | **0.508** | |

TPM: transcripts per million

*crh*: *corticotropin-releasing hormone*

*pomc*: *pro-opiomelanocortin*

Table S4 Changes of *dlx5, col1a1, and calcitonin* mRNA expression in the skin (pooled samples) of flounder reared in SSW and DOW.

| **TPM** | **SSW** | **DOW** |
| --- | --- | --- |
| ***dlx5*** | **2.43501** | **3.40523** |
| ***col1a1*** | **71.1460832** | **205.855033** |
| ***calcitonin*** | **1.304** | **2.940** |

TPM: transcripts per million

*col1a1*: *collagen type I alpha1*

Table S5 Trace mineral concentrations in the plasma of flounder rearing with SSW or DOW.

|  | **SSW** | **DOW** |
| --- | --- | --- |
| **Ba (mg/L)** | 5.74 | 4.72 |
| **V (mg/L)** | 114 | 97.3 |
| **Rb (μg/L)** | 22.9 | 18.4 |
| **Pb (μg/L)** | 2.74 | 2.31 |
| **Cu (μg/L)** | 37.4 | 374 |
| **Fe (μg/L)** | 464 | 341 |
| **Ni (μg/L)** | 38.8 | 35.8 |
| **Cr (μg/L)** | 13.1 | 12 |
| **Co (μg/L)** | 1.16 | 1.28 |

Table S6 Sequences of primers used for quantitative real-time PCR analysis.

Name Forward primer Reverse primer 　 Accession No.

*dlx5* GTTCTTCACCAACTCCAACCGAAC AATCGGGAGGTACTGGGTCTTC AB685218

*col1a1* TGCAACCAGGATGCCATCAA ATGAGGCGCAGGAAGGTGAG AB685219

*calcitonin* GCTCCAATCTCAGCAC GTCTGCGGGAAAGTTT　　 LC744804

*ef1a* ATTGTTGCTGGTGGTGTTGG 　　　 GGCACTGACTTCCTTGGTGA 　　 AB979720

*col1a1*: *collagen type I alpha 1, ef1α*: *elongation factor-1α*
